# Risk of dislocation using large- vs. small-diameter femoral heads in total hip arthroplasty

**DOI:** 10.1186/1756-0500-5-553

**Published:** 2012-10-05

**Authors:** Johannes F Plate, Thorsten M Seyler, D Alex Stroh, Kimona Issa, Michael Akbar, Michael A Mont

**Affiliations:** 1 Department of Orthopaedic Surgery, Wake Forest School of Medicine, Medical Center Boulevard, Winston-Salem, NC, 27157-1070, USA; 2 Department of Orthopaedic Surgery and Rehabilitation Medicine, University of Heidelberg, Schlierbacher Landstrasse 200a, Heidelberg, Germany; 3 Rubin Institute for Advanced Orthopedics, Center for Joint Preservation and Replacement, Sinai Hospital of Baltimore, 2401 West Belvedere Avenue, Baltimore, MD, 21215, USA

**Keywords:** Large-diameter, Dislocation, Total hip arthroplasty, Harris Hip score

## Abstract

**Background:**

Dislocation remains a difficult problem in total hip arthroplasty. Large-diameter femoral heads may lower the incidence of dislocation by enhancing the jump distance and decreasing impingement, but their performance against small-diameter heads has not been assessed. This study compared the mid-term radiographic and functional outcomes of two matched cohorts of patients undergoing total hip arthroplasty who had a high pre-operative risk for dislocation and who received either small-diameter (26- or 28-millimeters) or large-diameter (≥36-millimeters) femoral heads.

**Methods:**

All patients who received large-diameter heads (≥36-millimeter) between 2002 and 2005, and who had pre-operative risk factors for dislocation, were identified in the institution’s joint registry. Forty-one patients (52 hips) who received large-diameter heads were identified, and these patients were matched to 48 patients (52 hips) in the registry who received small-diameter femoral heads.

**Results:**

At mean final follow-up of 62 months (range, 49 to 101 months), both groups achieved excellent functional outcomes as measured by Harris Hip scores, with slightly better final scores in the large-diameter group (90 vs. 83 points). No patient showed any radiographic signs of loosening. No patient dislocated in the large-diameter femoral head group; the smaller-diameter group had a greater rate of dislocation (3.8%, 2 out of 52).

**Conclusions:**

Large-diameter femoral head articulations may reduce dislocation rates in patients who have a high pre-operative risk for dislocation while providing the same functional improvements and safety as small-diameter bearings.

## Background

Dislocation remains one of the most common complications following total hip arthroplasty (THA), and accounts for 17.5 to 22.5% of revision surgeries [1-7]. The overall incidence of dislocation after primary THA ranges from 0.7 to 5.8%, and may be as high as 20% or greater after revision THA [2,4,7-10]. Revision surgery due to dislocation after primary or revision THA accounts for $220 million annually in the Medicare population [6], and, as the number of operations performed increases, there will be an increased demand for component stability to sustain an active population. Most known patient risk factors for dislocation (shown in Table 1) cannot be easily changed by surgeons and, to reduce the risk, a number of modifications to traditional THA have been suggested [8,9,11-23].

**Table 1:** Risk factors and treatment options for THA dislocation

**Risk Factors**	**Treatment Options**
Alcoholism	Bipolar THA
Body mass index [11]	Constrained liners
Congenital hip dysplasia [8]	Large-diameter femoral heads
Dementia, Confusion, Psychosis [11,14,15,19,22]	Modular component exchange
Inflammatory arthritis [8,12,15,16,23]	Non-operative management (closed reduction, casts, braces)
Patient age (>80 years) [8,9,13,17,18]	Tripolar THA
Revision Surgery [16,19]	Trochanteric advancement
Posterolateral approach [16]	
*THA: total hip arthroplasty*	

Among the modifiable, implant-related risk factors for dislocation, increasing the distance of vertical displacement of the femoral head from the center of the acetabular component (jump distance), has demonstrated promise through the use of large-diameter femoral head components. Increasing jump distance prevents component-on-component or bone-on-bone impingement that may act as lever arm dislodging the femoral head from the acetabulum, and *in vitro* studies have demonstrated this change in jump distance using large-diameter femoral heads [24]. Several studies have shown reduced dislocation rates in experimental models, and after both primary and revision THA, through the use of large-diameter femoral heads (>36 mm) compared to both small-diameter components (26- or 28-millimeters) [25] or the use of constrained liners [26]. Other reports do not show the same success in improving stability [1,27] or suggest subtypes of patients who remain prone to dislocation. There are few reports that compare large-diameter femoral heads to small-diameter implants based on the clinical and radiographic outcomes after surgery [28-31].

Based on the variable outcomes of large-diameter femoral heads in the literature, we performed this study to understand our experience with large-diameter (>36 mm) and small-diameter (26- or 28-millimeters) femoral heads in patients at high risk for post-operative dislocation. The purpose of the present study was to compare the intra-operative results, as well as the clinical and radiographic outcomes, after THA in two similar cohorts of patients at high risk for post-operative dislocation who received either large-diameter (≥36-millimeter) or small-diameter (26- or 28-millimeter) femoral heads.

## Methods

An analysis of the joint registry at a single institution including all total hip arthroplasties done between 2002 and 2005 was performed to identify all patients who had received large-diameter femoral heads, had a high pre-operative risk of dislocation (one or more positive risk factors for dislocation, Table 1), and who had a minimum of 24 months follow-up. Large femoral head was defined as a diameter greater than or equal to 36 millimeters (median was 42 mm, range 36 mm to 50 mm). Forty-one patients (52 hips) were identified who had a mean age of 52 years (range, 30 to 84 years). The most common pre-operative diagnosis was osteoarthritis (63%, 33 out of 52 hips), followed by osteonecrosis (33%, 17 out of 52 hips). No patients were lost to follow-up. The demographic information for these patients can be found in Table 2. Patient demographic and clinical data, including pre-operative evaluations, labwork, imaging, intra-operative dictation, and post-operative evaluations, were reviewed from inpatient and clinic charts. Ethical approval for this study was obtained from the Institutional Review Board at Sinai Hospital of Baltimore, and informed consent was not required for patients to be included in this review.

**Table 2:** Patient demographics and statistical comparison

	**Large-diameter group**	**Small-diameter group**	p-Value*	***(95% Confidence interval)***
Hips (Patients)	52 (41)	52 (48)		
Age (range in years)	52 (30–84)	49 (31–83)	0.822	(−4.719; 3.758)
Gender				
*Male*	29 (56%)	29 (56%)	1	(−0.195; 0.195)
*Female*	23 (44%)	23 (44%)	1	(−0.195; 0.195)
Body mass index (kg/m^2^)	32.6 (21.3-58.7)	29.9 (18.6-58.9)	0.219	(−1.254; 5.415)
Alcoholism	8	5	0.374	(−0.072; 0.187)
Diagnosis				
*Osteoarthritis*	33 (63%)	26 (50%)	0.169	(−0.058; 0.327)
*Osteonecrosis*	17 (33%)	24 (46%)	0.163	(−0.325; 0.056)
*Hip dysplasia*	2 (4%)	2 (4%)	1	(−0.076; 0.076)
Follow-up months (range in months)	72 (58–110)	72 (50–86)	0.652	(−4.768; 2.998)

*p-value less than 0.05 in a 95% confidence interval was considered to be significant.

In a consecutive search of the institution’s joint registry, patients with large-diameter femoral heads were matched to a second group of patients who also had a pre-operative risk of dislocation and who received a femoral head that was either 26 or 28 millimeters in diameter (small-diameter femoral head). Patients were selected to match in age ± three years, gender, body mass index within three kg/m^2^, pre-operative diagnosis, length of follow-up within six months, and if one or multiple risk factors for dislocation were present pre-operatively. The mean age for this patient group was 49 years (range, 31 to 83 years) with a median femoral head size of 28 mm (range 26 mm to 28 mm). Demographic variables for these patients are also shown in Table 2. In no demographic variable was there found to be a significant difference between the two groups.

All procedures were performed using an antero-lateral approach by the senior author (MAM), with implantation of a proximally coated cementless prosthesis. Patients underwent identical post-operative rehabilitation. All patients were maintained at 50% weight-bearing for 6 weeks using crutches or a walker. After this time, they progressed to full weight-bearing. Supervised physical and occupational therapy was provided from weeks 6 to 10, which focused on gait training, range-of-motion, and strengthening exercises.

Patients were seen for follow-up visits at approximately 3 months, 6 months, 1 year, and annually thereafter. The mean follow-up time was 72 months (range, 58 to 110 months) for the large-diameter group and 72 months (range, 50 to 86 months) for the small-diameter group. Functional outcomes measured included pain, function, range-of-motion, and deformity, which were combined to give the Harris Hip score. Additionally, the rate of dislocation was recorded for both patient cohorts.

Radiographically, patients were monitored for the development of progressive radiolucencies, changes in implant alignment, or migration at each follow-up visit. Heterotopic ossification was classified in all patients according to Brooker et al. [32]. Both groups were reported on previously and now include longer follow-up and radiographic analysis [26].

Data was exported from the data registry into Excel spreadsheet format (Microsoft Office 2007, Microsoft Corporation, Redmond, Washington). Statistical analyses were performed with SPSS Statistics 17 (SPSS Inc., Chicago, Illinois) using Student *t*-test and chi-square analysis with an alpha of 0.05 to compare results between both groups.

## Results

In the large-diameter group, no patient sustained a dislocation of the implanted hip, compared to 2 patients (2 hips, 3.8%) in the small-diameter group who sustained dislocations and required revision surgery (Table 3). One of these instances was in a morbidly obese patient who dislocated one month after surgery and was subsequently revised to a constrained acetabular liner. At a final follow-up of 72 months, this patient was pain free with full functional range-of-motion in the affected hip and no further complications. The other patient dislocated a total of three times following the first two months after surgery. The patient was reduced closed under fluoroscopic guidance after the first two dislocations. After the third dislocation the patient underwent revision surgery with the placement of a constrained liner. At a final follow-up of 84 months, this patient was doing well without further complications and was pain-free with no functional limitations in the affected hip.

**Table 3:** Patient clinical and radiographic outcomes

	**Large-diameter group**	**Small-diameter group**	p-Value*	***(95% Confidence interval)***
Harris Hip Score (points)				
*Pre-operative*	32 (7–75)	30 (3–61)	0.374	(0.140; 13.352)
*Post-operative*	90 (50–100)	83 (7–100)	0.005	(0.140; 13.352)
Cup inclination (degrees)	35.9 (20–50)	37.7 (24–58)	0.236	(−4.918; 1.233)
Femoral offset (mm)				
*Pre-operative*	44.0 (21–63)	48.4 (25–58)	0.054	(−8.914 to 0.073)
*Post-operative*	52.5 (39–73)	53.9 (37–71)	0.478	(−5.273 to 2.498)
Dislocations	0 (0%)	2 (3.8%)	0.495	

*p-value less than 0.05 in a 95% confidence interval was considered to be significant.

At final follow-up, the mean Harris Hip score for the large-diameter group had improved from 32 points (range, 7 to 75 points) to 90 points (range, 50 to 100 points) (Table 3). This was significantly greater (p = 0.045) than the final score obtained in the small-diameter group, who improved from a mean of 30 points (range, 3 to 61 points) to 83 points (range, 70 to 100 points). Patients in both groups were found to have similar range-of-motion, except for internal rotation of the hip which was found to be a mean 5 degrees greater in the large-diameter group (p = 0.002).

Radiographically, the mean cup inclination angle was 35.9 degrees (range, 20 to 50 degrees) for the large-diameter group, compared to 37.7 degrees (range, 24 to 58 degrees) in the small-diameter group (p = 0.236). The femoral offset was changed from a mean of 48 mm (range, 25 to 58 mm) preoperatively to 54 mm (range, 37 to 71 mm) postoperatively in the small-diameter group (p < 0.001). In the large-diameter group, the femoral offset changed from a mean of 44 mm (range, 21 to 63 mm) to 53 mm (range, 39 to 73 mm, p < 0.001). There was no difference in femoral offset between both groups (p = 0.478). In all patients, leg length was restored appropriately. Similar amounts of heterotopic ossification were found in both groups, and there were no progressive radiolucencies noted.

## Discussion

Dislocation after primary and revision THA remains a devastating complication with a high economic burden and impact on hospital resources [6]. While the overall outcomes of primary THA are excellent with greater than 95% survivorship at long-term follow-up, patients with a high dislocation risk may benefit from a prosthesis that improves stability and reduces the chance of revision surgery. In this study, two patient cohorts with similar pre-operative functional scores and risk profiles received either large-diameter or small-diameter heads. Although both groups achieved excellent results, we noted no dislocations and improved functional hip scores in the large-diameter group compared to the small-diameter group, which had two dislocations (3.9%). Radiographic findings were similar between groups.

Pre-operative evaluation of primary THA patients should include a careful stratification of risk factors for dislocation. Both patients in the small-diameter group may have benefitted from a large-diameter femoral head. However, large-diameter metal-on-metal femoral heads had just become available prior to the study period and limited experience with these devices was available. The use of large-diameter femoral heads conventional metal-on-polyethylene articulation has been associated with higher wear rates. Though concerns about metal ion debris have been raised with metal-on-metal articulation [33], survivorship of these bearings was found to be approximately 95% after mean follow-up between 3 and 10 years [34]. The results of this study suggest that large-diameter metal-on-metal femoral heads may be a suitable treatment for patients at high-risk of dislocation. Our strategy for selecting the appropriate size femoral component included careful pre-operative evaluation and stratification of risk factors for dislocation in every primary total hip arthroplasty patient. Should the patient have one risk factor for dislocation (e.g. age older than 80, higher body mass index, inflammatory arthritis, etc.) implantation of a large-diameter component is considered and discussed with the patient. If patients are found to have two or more risk factors for dislocation, large-diameter implants have become the treatment of choice. All of these procedures were performed through anterolateral approach and the bias through surgical approach was minimized for these two patient cohorts. All of the dislocations in the small-diameter group had occurred during the first 2 months post-operative period which can mainly be considered implant related compare to late failures that can be due to other factors such as soft tissue compromise, implant migration, neurologic disorder, etc*.* The results of this study suggest that large-diameter femoral heads may be a suitable treatment for patients who are at risk for dislocation.

There are several limitations to the present study. These results represent only short-term follow-up, and longer follow-up is necessary for confirmation. The retrospective study design is not as powerful as a randomized control trial, and such studies will be necessary in the future. All procedures were performed using an antero-lateral approach, which has been reported to decrease the risk for post-operative dislocation [35]. Dudda *et al. *[35] found a 6-fold increased risk for dislocation when the posterior approach was used compare to an anterolateral or straight lateral approach, or a lateral approach with trochanteric osteotomy. In contrast, Ho *et al*. [36] and Hummel *et al. *[37] reported a decrease in dislocation rates after primary or revision surgery using a posterior approach when careful capsular repair was performed. While the influence of surgical approach on dislocation rates following THA requires further investigation, in our study, the use of same surgical approach in all patients would have minimized any potential bias in this regard. This study aimed at assessing the influence of femoral head diameter on dislocation rates in patients who were at high risk for postoperative dislocation. However, we do agree that a larger sample size and longer follow-up of all patients would be of benefit and may be performed in the future.

The findings of this study agree with previous reports that show excellent results in avoiding dislocation with large-diameter femoral heads. Cuckler *et al*. [28] reported no early dislocations out of 616 arthroplasties performed using a 38-millimeter diameter femoral head, compared with 2.5% out of the 78 patients who received 28-millimeter diameter heads. These findings are, however, limited to the first 3 months following surgery. Peters *et al. *[30] compared dislocation rates between 136 patients who received 38-millimeter heads via posterior approach, and 160 patients who received 28-millimeter heads via Hardinge approach. At a mean follow-up of 52 months, there were no dislocations in the 38-millimeter group, compared to four in the 28-millimeter group. In a retrospective analysis of 230 patients who received either 28- or 36-millimeter femoral head components following revision arthroplasty for instability, Kung *et al. *[38] showed that larger femoral heads lowered dislocation rates from 12.7 to 0% when the abductor mechanism was present and from 40 to 33% when abductors where absent. Garbuz *et al. *[39] randomized 184 patients who underwent revision THA to either a 32-millimeter diameter femoral head, or either a 36- or 40-millimeter femoral head. At a final follow-up of 5 years (range, 2 to 7 years), the dislocation rate in the larger diameter group was 1.1% (one out of 92 patients) compared to 8.7% (eight out of 92 patients) in the small-diameter group. Stuchin [6] reported no dislocation in a short-term follow-up evaluation of thirty-four patients who had undergone forty total hip arthroplasties with use of a modular metal-on-metal articulation with an anatomic diameter femoral head. They reported four these patients were profoundly disabled and had bone or soft-tissue deficiencies that could have increased the risk for dislocation. He concluded anatomic diameter femoral heads may offer distinct theoretical advantages in total hip arthroplasty. Lombardi *et al. *[29] reviewed 1748 patients (2020 hips) who underwent THA with femoral heads 36 mm in diameter or larger at their institution between 2001 and 2008. At a mean follow-up of 31 months (range, 1 to 102 months) only one dislocation had occurred (0.05%). At total of 379 hips (18.8%) were deemed to be at risk for dislocation pre-operatively, and none of these patients sustained a dislocation. Bistolfi *et al. *[40] compared the dislocation rates between 198 consecutive conventional THAs with 28 mm femoral heads and 259 consecutive conventional THAs with 36 mm femoral heads. During the first year after THA, there 6 dislocations in the 28 mm group compared to one dislocation in the 36 mm group (p = 0.046) with a higher risk ratio for dislocation (7.85, p = 0.046) for the 28 mm group.

This study contrasts with other reports that show higher rates of revision and worse clinical outcomes with large-diameter femoral heads. Amstutz *et al.*[1] reported on 135 patients (140 hips) who had a mean age 62 years (range, 16 to 95 years) and who were implanted with large-diameter (≥36-millimeter) heads either for recurrent dislocation, revision unrelated to dislocation, or primary operation. At a mean follow-up of 5.5 years (range, 1 to 17 years), 119 patients (124 hips) were stable without recurrent dislocations and 16 patients (11%) remained unstable and subsequently underwent re-revision. However, only 6 of these patients were unstable due to recurrent dislocation, and in this group all 6 patients were found to have loose acetabular components thought to be responsible for the dislocation. Berton *et al*. [27] studied 92 patients (100 hips) who had a mean age of 50 years (range, 18 to 70 years) and who received large-diameter femoral head prostheses during THA. At a final follow-up of 5 years, there were no cases of dislocation, but the reported survival rate was 92.4%. Although these failures were attributed to difficulty in orienting the component and poor porous coating, it should be noted that this represents early experience with large-diameter prostheses that has recently improved.

## Conclusions

Although dislocation remains a troublesome complication of total hip arthroplasty, the use of large-diameter femoral heads can aid in increasing stability and reducing the risk of revision surgery. The authors recommend that large-diameter femoral heads be considered in patients with pre-operative risk factors for dislocation (low or high risk factors). Specifically, for morbidly obese patients and patients undergoing revision surgery for recurrent dislocations, large-diameter femoral heads may be beneficial, while providing improved functional outcomes compared to small-diameter bearings.

## Abbreviation

THA: Total hip arthroplasty.

## Competing interests

MAM has received funding from Stryker Orthopaedics and Wright Medical Technology. Funding was received in support of this work. The authors note no other financial or non-financial competing interests.

## Authors’ contributions

JFP, TMS – Patient chart review, data collection, assembly of study group, manuscript preparation. MA – data review, statistical analysis. DAS – manuscript preparation; KI - manuscript preparation and revisions; MAM – surgical attending, manuscript preparation. All authors have read and approved the final manuscript.
